# Changes in oncological surgical volume and postoperative survival during the COVID-19 pandemic: a retrospective multicenter study in a regional German hospital network

**DOI:** 10.3389/fonc.2026.1931801

**Published:** 2026-09-07

**Authors:** Antonia L. Stengler, Jörg Kleeff, Kerstin Lorenz, Nadja Weigert, Sandra Adam, Simon Rieder, Ulrich Ronellenfitsch

**Affiliations:** 1 Department of Visceral, Vascular and Endocrine Surgery, University Hospital Halle (Saale) and Medical Faculty of the Martin-Luther-University Halle-Wittenberg, Halle (Saale), Germany; 2 BG Klinikum Bergmannstrost Halle (Saale), Department of General, Visceral and Vascular Surgery, Halle (Saale), Germany; 3 Department of General and Visceral Surgery, Diakonie Hospital Halle, Halle (Saale), Germany

**Keywords:** cancer surgery, COVID-19 pandemic, pandemic preparedness, postoperative mortality, surgical volume

## Abstract

**Purpose:**

The COVID-19 pandemic led to delays in elective procedures and a reduction in oncological resections worldwide, potentially affecting the prognosis of cancer patients. This study assessed changes in surgical volume, tumor stage distribution, and postoperative overall survival across the pre-pandemic, pandemic, and post-pandemic periods within a regional network comprising one tertiary referral center and two peripheral hospitals.

**Methods:**

This retrospective multicenter observational study included oncological surgical procedures performed in three surgical departments in Halle (Saale) between August 1, 2017, and April 30, 2025. The data were stratified into pre-pandemic, pandemic, and post-pandemic periods. Study outcomes included surgical volume, tumor stage distribution, and postoperative overall survival. Survival analyses were performed in a patient-based cohort using the first eligible procedure as the index operation and assessed at predefined postoperative time points.

**Results:**

A total of 2871 oncological surgical procedures were analyzed across pre-pandemic (n=952), pandemic (n=940), and post-pandemic (n=979) periods. Monthly surgical volume fluctuated across the observation period, reaching its lowest value in April 2020 and December 2021 (n = 20) and its highest value in January 2021 (n = 40). Among patients included in the survival analysis, the proportion of UICC stage IV was higher during the pandemic (26.8%) than in the pre-pandemic (23.9%) or post-pandemic (24.2%) periods. Kaplan–Meier analysis showed a trend toward shorter postoperative overall survival during the pandemic period (log-rank p = 0.066). In multivariable Cox regression adjusted for age, sex, tumor stage, and resection site, the pre-pandemic cohort was associated with a lower hazard of death than the pandemic cohort (HR 0.78, 95% CI 0.65-0.93; p = 0.005).

**Conclusion:**

Changes in oncological surgical volume were observed across the study periods. During the pandemic period, a higher proportion of advanced tumor stages at presentation and a trend toward shorter postoperative overall survival were observed. After adjustment for baseline characteristics, the pre-pandemic cohort showed a lower mortality hazard than the pandemic cohort. These findings support the importance of resilient healthcare infrastructures for continuous, guideline-concordant oncological care.

## 1 Introduction

The COVID-19 pandemic posed significant challenges to healthcare systems worldwide. In order to allocate healthcare resources for the treatment of patients with COVID-19, hospitals in Germany and worldwide were instructed to postpone selected elective procedures. This measure resulted in a significant reduction in oncological resections (1). An analysis of gastrointestinal oncological procedures, including resections of the esophagus, stomach, liver, pancreas, colon, and rectum, showed a 7.4% reduction in monthly cancer surgeries in Germany in 2020. This decline was especially pronounced during the lockdown phases. Furthermore, a significant inverse correlation was observed between regional SARS-CoV-2 incidence rates and resection numbers (2).

Oncological patients often require prolonged hospital stays and, in certain cases, intensive care unit (ICU) admission during their surgical treatment (3). The surge in COVID-19 cases led to peak occupancy of ICUs in Germany and Saxony-Anhalt in January 2021, which placed additional pressure on hospital capacities and potentially affected the organization of oncological care (4). For patients with malignant tumors, it is recommended to initiate diagnostic and therapeutic measures without undue delays. Specifically, treatment intervals of more than four weeks between diagnosis and the start of therapy should be avoided (5–8), as longer waiting times are associated with unfavorable outcomes. These include an increased risk of tumor progression and metastasis, as well as a deterioration in both short- and long-term survival rates and quality of life (1, 7, 9). However, reductions in surgical volume should be interpreted separately from delays in diagnosis or treatment, as the number of performed procedures alone does not directly reflect healthcare access or unmet surgical need.

Although reductions in oncological surgical activity during the COVID-19 pandemic have been reported, few studies have simultaneously evaluated regional surgical activity, tumor stage at presentation, and postoperative overall survival across the pre-pandemic, pandemic, and post-pandemic periods within the same healthcare network.

The aim of this study was to assess changes in oncological surgical volume, tumor stage distribution at the time of presentation, and postoperative overall survival across these three periods in a regional multicenter hospital network. We hypothesized that these outcomes differed between the pre-pandemic, pandemic, and post-pandemic periods.

## 2 Methods

For this retrospective multicenter observational study, all gastrointestinal and endocrine oncological surgeries from the department of General Surgery and Visceral, Vascular and Endocrine Surgery and two affiliated hospitals in Halle (Saale), Saxony-Anhalt, Germany, between August 1, 2017, and April 30, 2025, were evaluated. All participating institutions are regional centers, have a broad surgical spectrum, and are among the central healthcare providers in the region. While two of the hospitals provide primary oncological gastrointestinal surgery (mostly colorectal resections), the third hospital serves as a secondary and tertiary referral center and offers more complex oncological gastrointestinal and endocrine surgeries (e.g., hepatectomy, pancreatectomy, esophagectomy, adrenalectomy). Data collection was performed using the hospitals’ information systems, with all surgeries documented in the theater schedule being included. Data extraction was performed manually based on predefined inclusion criteria. Completeness and plausibility checks were performed by reviewing the original surgical documentation.

The observation period was divided into the pre-pandemic period (August 1, 2017 – February 29, 2020), the pandemic period (March 1, 2020 – September 30, 2022), and the post-pandemic period (October 1, 2022 – April 30, 2025). In Germany, the COVID-19 pandemic was characterized by six distinct waves, defined based on national infection patterns. The first wave lasted from March 1 to May 31, 2020, encompassing the first nationwide lockdown from March 16 to May 3, and was followed by a summer plateau from June 1 to September 30, 2020. The second wave extended from October 1, 2020, to February 28, 2021, including the second lockdown from November 2, 2020, to January 10, 2021. The third wave occurred from March 1 to May 31, 2021, followed by a summer plateau from June 1 to September 30, 2021. The subsequent fourth, fifth, and sixth waves were observed from October to December 2021, January to May 2022, and June to September 2022 (10). All three periods had an identical duration of 31 months to ensure comparability.

All patients with histologically confirmed malignant tumors (ICD-10: C00–C97, D00–D09, D37–D48) who underwent surgery within the aforementioned surgical disciplines, i.e., gastrointestinal and endocrine oncological surgery, were included. Both curative and palliative procedures were considered, provided they primarily aimed at resecting the primary tumor or metastatic deposits. Surgeries outside the specified surgical disciplines (e.g., neurosurgical procedures), as well as procedures on tumor patients without tumor resection, such as port implantations, stoma placements, and wound care, were excluded. Cases where a benign histological finding was confirmed despite a preoperative suspicion of malignancy were also excluded.

Patients could be included multiple times if they underwent more than one procedure during the study period. For survival analyses, a patient-based approach was applied and only the first eligible procedure during the study period was considered as the index operation. Subsequent procedures were not included in the survival analysis. Simultaneous resections involving multiple anatomical sites were classified according to the primary oncological resection site documented in the surgical report. If duplicate patient records across participating hospitals could be identified, only the first eligible procedure was retained.

The analysis was conducted in a fully anonymized manner, ensuring that individual patients could not be identified.

Resection sites with fewer than 75 procedures across the entire study period were combined into an “other” category to avoid very small subgroup sizes and unstable statistical estimates. These included: appendix, duodenum, gallbladder, ileum, liver, thymus, and soft tissue sarcoma. This category was created solely for statistical purposes and should not be interpreted as a clinically homogeneous tumor group.

In addition, Union for International Cancer Control (UICC) stage, resection status, American Society of Anesthesiologists (ASA) classification, surgical intent, and surgical urgency were assessed. Resection status was defined as follows: R0 indicated complete tumor removal with no microscopic or macroscopic residual tumor at the resection margins. R1 denoted the presence of microscopic residual tumor at the resection margins, while R2 referred to macroscopically visible residual tumor. RX indicated an uncertain resection status, meaning that it was not possible to definitively determine whether the tumor had been completely removed (11). Tumors were staged according to the UICC classification system, ranging from stage 0 (no detectable tumor) to stage IV (metastatic) (12). The ASA grade is a classification system used to assess the preoperative health status of a patient and is known to closely correlate with the perioperative risk of complications. It ranges from ASA I (healthy patient, very low risk) to ASA V (moribund patient, very high risk) (13). Follow-up data were collected up to December 31, 2025. Hospital records were reviewed for patient follow-up visits, and additional information was obtained from the Krukenberg Cancer Center, which routinely contacts patients’ general practitioners and health insurance providers at regular intervals to update records on the most recent recurrence and vital status. The median follow-up duration was 20 months (IQR 3–55) for patients from the pre-pandemic, 19 months (IQR 4–41) for patients from the pandemic, and 8 months (IQR 1–15) for patients from the post-pandemic period. The shorter follow-up in the post-pandemic cohort was due to administrative censoring at the predefined end of the study period.

Statistical analysis was performed using IBM SPSS Statistics version 31 (IBM Corp., Armonk, N.Y., USA). As this was a retrospective study including all eligible oncological surgical procedures performed during the predefined study period, no formal sample size calculation was performed. The study was reported according to the Strengthening the Reporting of Observational Studies in Epidemiology (STROBE) guidelines for cohort studies. The completed STROBE checklist is provided as **Supplementary Material S1**.

Postoperative overall survival was estimated using the Kaplan-Meier method, and differences between the time periods were assessed using the log-rank test. Survival rates were reported at one, six, 12, 24 and 36 months after surgery. This time-based stratification enables a differentiated assessment of short-, intermediate-, and long-term survival. Due to incomplete follow-up in the post-pandemic cohort, survival rates for this group were only reported for one and six months after surgery.

To assess the association between study period and overall survival, a univariate Cox regression was conducted. This was followed by a multivariable Cox regression to evaluate the association between study period and survival while adjusting for clinically relevant variables. Only variables with sufficient clinical relevance and data completeness (greater than 80%) were included in the multivariable model to minimize potential bias. The multivariable model was adjusted for age, sex, tumor stage, and resection site to account for potential differences in baseline characteristics and tumor entity distribution between study periods. All hospital- and resection site-specific analyses were considered exploratory and were performed for descriptive purposes only. No adjustment for multiple testing was applied.

The Ethics Committee of the Faculty of Medicine at the University of Halle reviewed the study protocol and waived the requirement for formal ethics approval because of the retrospective design and the use of anonymized data (Reference number 2025-223).

## 3 Results

### 3.1 Baseline characteristics

During the study period, a total of 2,871 oncological gastrointestinal and endocrine surgical procedures were performed, including 952 in the pre-pandemic period, 940 during the pandemic, and 979 in the post-pandemic period. Overall, 1,612 procedures (56.1%) were performed in male patients and 1,259 (43.9%) in female patients. The median age of the total cohort was 66 years (interquartile range [IQR] 56–76). Among procedures with available UICC stage, the majority of procedures in the pre- and post-pandemic periods were performed in patients with UICC stage I (27.5% and 29.1%), whereas during the pandemic period, the highest proportion of surgeries was observed in patients with UICC stage IV (26.8%). Across all periods, resections were predominantly performed with negative margins (R0 resection rate: 84.1%). Among patients with available ASA classification (n=2129), which was only routinely documented at the tertiary referral center, most were classified as ASA physical status III (61.8%), indicating severe systemic disease (13). The majority of procedures were conducted for resection of the primary tumor (n = 2,718; 94.7%), while 153 procedures (5.3%) were performed for metastatic disease. Surgical interventions were predominantly elective (98.2%), with emergency procedures accounting for only 1.8%. The thyroid (26.0%) and colon (21.7%) were the most frequent resection sites (**Table 1**).

**Table 1:** Characteristics of oncological surgical procedures and included patients.

Variable	Pre-pandemic period (n=952)	Pandemic period (n=940)	Post-pandemic period (n=979)	Total (n=2871)
Sex (%)				
male	544 (57.1)	529 (56.3)	539 (55.1)	1612 (56.1)
female	408 (42.9)	411 (43.7)	440 (44.9)	1259 (43.9)
Age				
median (IQR)	67 (57-77)	66 (56-76)	65 (55-74)	66 (56-76)
UICC stage (%)	n=849	n= 852	n=900	n=2601
0	8 (0.9)	13 (1.5)	15 (1.7)	36 (1.4)
I	233 (27.5)	217 (25.5)	262 (29.1)	712 (27.4)
II	190 (22.4)	206 (24.2)	214 (23.8)	610 (23.4)
III	215 (25.3)	188 (22.0)	191 (21.2)	594 (22.8)
IV	203 (23.9)	228 (26.8)	218 (24.2)	649 (25.0)
resectability (%)	n=850	n=854	n=878	n=2582
R0	716 (84.2)	724 (84.8)	731 (83.3)	2171 (84.1)
R1	84 (9.9)	91 (10.7)	103 (11.7)	278 (10.8)
R2	20 (2.4)	14 (1.6)	16 (1.8)	50 (1.9)
RX	30 (3.5)	25 (2.9)	28 (3.2)	83 (3.2)
ASA classification (%)^1^	n=669	n=725	n=735	n=2129
I	32 (4.8)	20 (2.8)	24 (3.3)	76 (3.6)
II	204 (30.5)	203 (28.0)	242 (32.9)	649 (30.5)
III	404 (60.4)	467 (64.4)	444 (60.4)	1315 (61.8)
IV	28 (4.2)	34 (4.7)	24 (3.3)	86 (4.0)
V	1 (0.1)	1 (0.1)	1 (0.1)	3 (0.1)
Surgical intent				
primary tumor resection	899 (94.4)	896 (95.3)	933 (95.3)	2728 (95.0)
metastasectomy	53 (5.6)	44 (4.7)	46 (4.7)	143 (5.0)
Surgical urgency				
elective	930 (97.7)	923 (98.2)	967 (98.8)	2820 (98.2)
emergency	22 (2.3)	17 (1.8)	12 (1.2)	51 (1.8)
Resection site (%)				
colon	234 (24.6)	192 (20.4)	196 (20.0)	622 (21.7)
stomach	60 (6.3)	52 (5.5)	48 (4.9)	160 (5.6)
esophagus	54 (5.7)	73 (7.8)	73 (7.5)	200 (7.0)
pancreas	109 (11.4)	101 (10.7)	113 (11.5)	323 (11.2)
rectum	143 (15.0)	135 (14.4)	105 (10.7)	383 (13.3)
thyroid	213 (22.4)	233 (24.8)	302 (30.8)	748 (26.0)
other^2^	86 (9.0)	110 (11.7)	96 (9.9)	292 (10.2)
liver metastases	53 (5.6)	44 (4.7)	46 (4.7)	143 (5.0)

^1^ ASA classification was available only for Hospital 1, as this variable was not routinely documented in the electronic systems of Hospitals 2 and 3 during the study period. ²other: appendix, duodenum, gallbladder, ileum, liver, thymus, and soft tissue sarcoma. Percentages are based on available data. Denominators are provided for variables with missing values. Pre-pandemic: 01.08.2017–29.02.2020; pandemic: 01.03.2020–30.09.2022; post-pandemic: 01.10.2022–30.04.2025. ASA, American Society of Anesthesiologists; IQR, interquartile range; UICC, Union for International Cancer Control.

A hospital-stratified presentation of baseline characteristics is provided in **Supplementary Table 2**.

### 3.2 Surgical volume during the pandemic period

**Figure 1:**
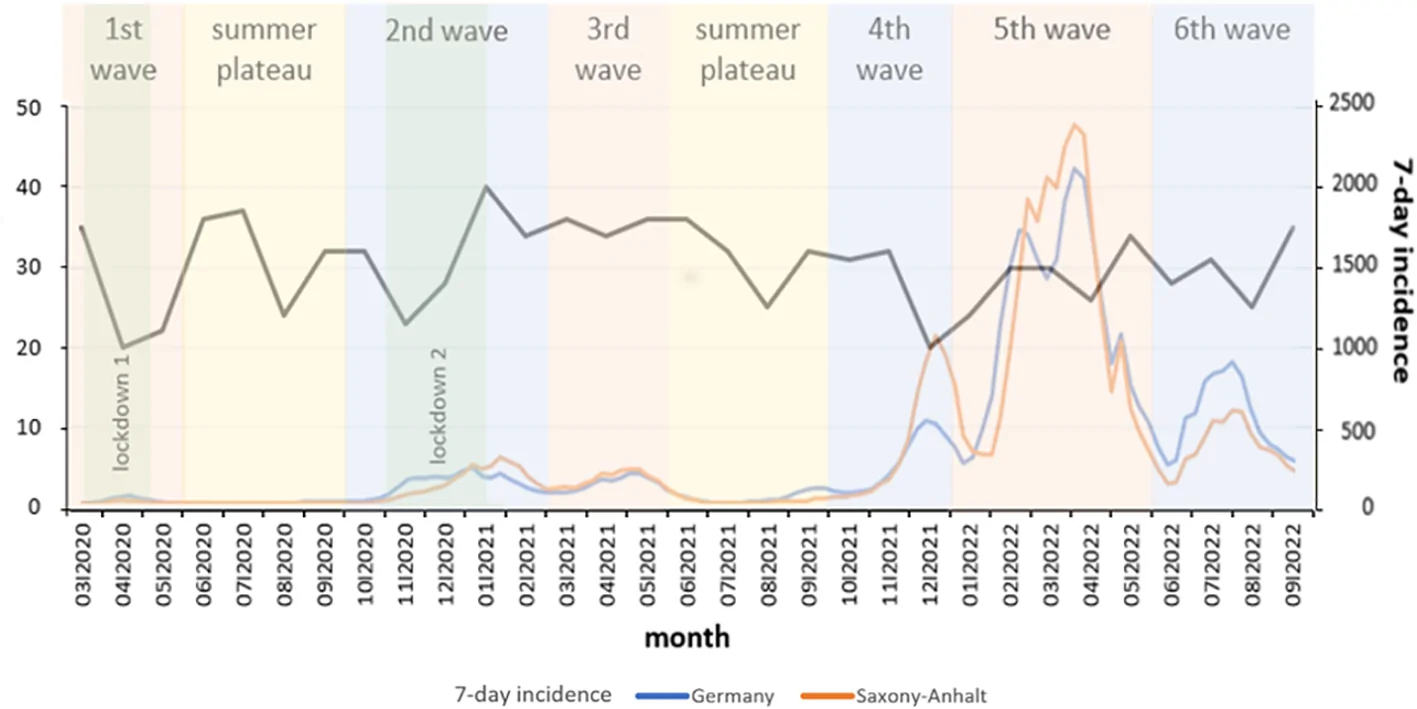
Monthly surgical volume for oncological gastrointestinal and endocrine operations during the pandemic in relation to the 7-day incidence per 100,000 inhabitants in Germany and Saxony-Anhalt, and waves of the COVID-19 pandemic in Germany. Incidence data were obtained from publicly available surveillance reports (10).

**Figure 1** illustrates the monthly surgical volume during the pandemic, stratified according to the six respective pandemic phases and summer plateaus (10). In the background, the 7-day incidence rates per 100,000 inhabitants for Germany and Saxony-Anhalt are depicted. Monthly surgical volume fluctuated throughout the observation period. The highest monthly number of procedures (n = 40) was recorded in January 2021 during the second wave of the pandemic, including the period of the second lockdown. In contrast, the lowest monthly number of surgeries was observed in April 2020 during the first wave and first lockdown, as well as in December 2021 during the fourth wave (n = 20).

### 3.3 Postoperative survival

**Figure 2:**
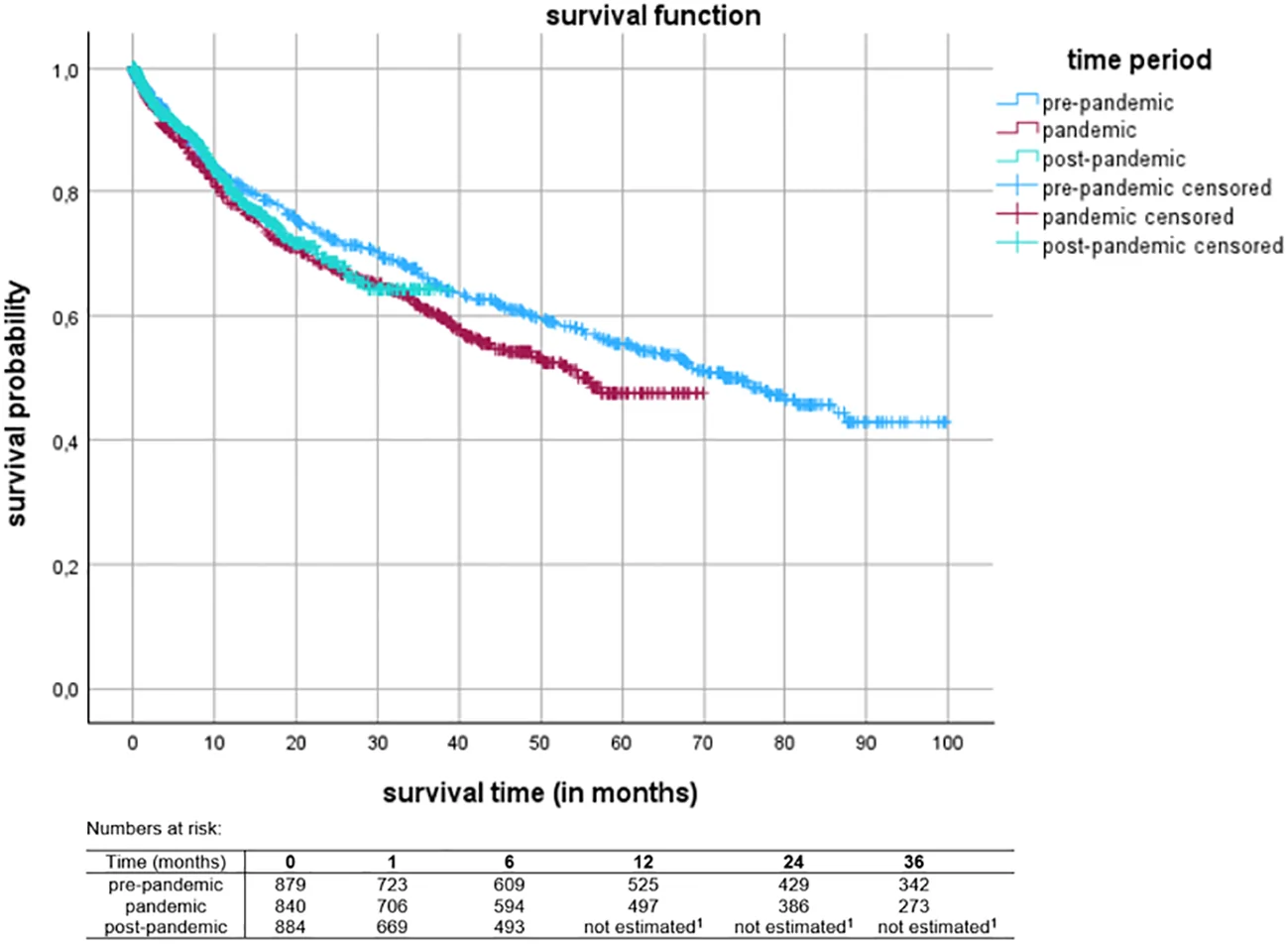
Kaplan-Meier survival curves stratified by period. pre-pandemic *(01.08.2017-29.02.2020)* n= 879; pandemic *(01.03.2020-30.09.2022)* n= 840; post-pandemic *(01.10.2022-30.04.2025)* n= 884. Differences between groups were assessed by log-rank test (p=0.066). ^1^follow-up pending.

The survival analysis was based on patient-level data, with each patient treated as an independent unit of observation. Overall, 172 patients underwent more than one eligible oncological procedure during the study period. The analysis included a total of 2,603 patients. During follow-up, 292 deaths occurred in the pre-pandemic cohort, 283 in the pandemic cohort, and 145 in the post-pandemic cohort. The Kaplan–Meier curves showed a trend toward differences between the study periods (log-rank p = 0.066). At the beginning of the observation period, the survival curves largely overlapped. From approximately 10 months postoperatively onwards, a separation of the pre-pandemic curve from the pandemic and post-pandemic curves became apparent. The latter two curves remained largely overlapping until around 30 months postoperatively, when maximum follow-up for patients operated in the post-pandemic period was reached (**Figure 2**).

The Kaplan–Meier survival probabilities at predefined time points are presented in **Table 2**. At one month, the survival probability across the three study periods was approximately 97%. After six months, it varied between 87.8% and 89.9%, and after 12 months, it was between 78.1% and 81.9%. After 24 months, the survival probability fell within the range of 68.1% to 72.8%, and after 36 months, it was between 61.0% and 66.1%. At the reported follow-up time points, survival probabilities in patients operated during the pandemic period were lower than those in the pre-pandemic and post-pandemic periods. Postoperative survival analyses, stratified by resection site and hospital, are presented in **Supplementary Tables 3**-**S13** and **Supplementary Figures 1**-**S11**. Kaplan–Meier analyses indicated a trend toward shorter survival in rectal cancer patients operated on during the pandemic period (p = 0.054).

**Table 2:** Kaplan–Meier survival probabilities at predefined postoperative time points for patients in the pre-pandemic, pandemic, and post-pandemic periods.

Postoperative time point	Pre-pandemic (n=879)	Pandemic (n=840)	Post-pandemic (n=884)
1 month	97.2%	97.1%	97.2%
6 months	89.5%	87.8%	89.9%
12 months	81.9%	78.1%	not estimated^1^
24 months	72.8%	68.1%	not estimated^1^
36 months	66.1%	61.0%	not estimated^1^

pre-pandemic (01.08.2017-29.02.2020); pandemic (01.03.2020-30.09.2022); post-pandemic (01.10.2022-30.04.2025); ^1^follow-up pending.

In the univariate Cox regression analysis, the hazard ratio (HR) for the pre-pandemic cohort was 0.82 (95% CI 0.69–0.97; p = 0.020), while the post-pandemic cohort had an HR of 0.92 (95% CI 0.75–1.13; p = 0.406), both compared to the pandemic cohort. In the multivariable model, adjusted for age, sex, tumor stage and resection site, the HR for the pre-pandemic cohort was 0.78 (95% CI 0.65–0.93; p = 0.005), and for the post-pandemic cohort, it was 0.94 (95% CI 0.76–1.16; p = 0.572) (**Table 3**).

**Table 3:** Univariate and multivariable Cox regression analysis of overall survival.

Time period	Unadjusted HR (95% CI)	p value	Adjusted^1^ HR (95% CI)	p value
pandemic (ref.)	1.00	–	1.00	–
pre-pandemic	0.82 (0.69-0.97)	0.020	0.78 (0.65-0.93)	0.005
post-pandemic	0.92 (0.75-1.13)	0.406	0.94 (0.76-1.16)	0.572

n= 2603 (univariate analysis); n= 2339 (multivariable analysis). Number of events included in the Cox regression: 720 in the univariate analysis and 680 in the multivariable analysis. Global model significance was assessed using the likelihood ratio test (χ² = 463.082, df = 14, p < 0.001).^1^Adjusted for tumor stage, resection site, sex and age. HR, hazard ratio; CI, confidence interval; ref., reference group.

As a sensitivity analysis, a 12-month landmark Cox regression was performed in the post-pandemic cohort. The hazard ratio estimates differed only minimally from those of the primary 6-month landmark analysis. The unadjusted HR was 0.87 (95% CI, 0.71–1.07; *p* = 0.194), and the adjusted HR was 0.90 (95% CI, 0.73–1.11; *p* = 0.321).

## 4 Discussion

This study analyzed changes in oncological surgical care during the COVID-19 pandemic for gastrointestinal and endocrine cancers in three collaborating hospitals in Saxony-Anhalt over a period encompassing a pre-pandemic, pandemic, and post-pandemic phase. Overall, surgical volumes were largely maintained throughout the study period, with only slight reductions observed during the pandemic, accompanied by phase-dependent fluctuations as well as changes in patient characteristics and survival outcomes.

The temporary decline in surgical volumes observed in this study, particularly during the early lockdown phases of the pandemic, can be reasonably explained by structural and health policy-related restrictions. According to an official resolution by the German federal government and the federal states on 12 March 2020, hospitals were recommended to postpone elective surgical procedures in order to increase healthcare capacity and ensure adequate intensive care resources for patients with COVID-19 (14). This measure led to a significant reduction in elective surgical procedures (15). In addition, staffing constraints further limited surgical activity. These findings are supported by Stöß et al., who observed a significant decrease in both oncological diagnoses and surgical treatments, particularly during the first wave of the pandemic (16). In contrast, the increase in surgical volumes observed in later phases of the pandemic in this study points to what is known as catch-up effects, where previously delayed surgeries were carried out once regular healthcare operations resumed. The temporary increase in surgical volume observed in January 2021, despite high SARS-CoV-2 incidence and increased ICU occupancy, likely reflects the continuation of prioritized oncological surgical pathways and institutional adaptations that allowed cancer surgery to proceed during later pandemic phases. As elective oncological procedures were not uniformly suspended throughout the pandemic, monthly variations may also reflect scheduling effects, deferred procedures from previous months, and differences in case distribution over time. Similar findings have been reported in recent studies, which show at least partial compensation for missed surgeries after restrictions were eased (17, 18). The renewed decline in surgical volumes during later pandemic waves coincided with periods of increased SARS-CoV-2 incidence, suggesting that surgical activity remained sensitive to pandemic-related healthcare pressures (19).

Another key finding of this analysis was the higher proportion of patients with UICC stage IV observed during the pandemic period. This result is clinically relevant, as later tumor stages are associated with poorer prognoses and more limited treatment options (20). This development may reflect multiple factors, including changes in referral patterns, case-mix, and healthcare utilization during the pandemic. However, the present study was not designed to evaluate diagnostic or treatment delays directly.

The literature shows that the COVID-19 pandemic was associated with a decline in cancer screening and delays in diagnosis. Zhang et al. demonstrated in a population-based analysis that interruptions in screening programs and diagnostic delays increase the risk of detecting tumors at more advanced stages (21). Additionally, recent international data reported substantial disruptions in cancer diagnosis during the pandemic, including a higher proportion of advanced-stage disease at presentation, which is consistent with the higher proportion of UICC stage IV tumors observed during the pandemic period in the present study (22). Furthermore, studies have shown that during the pandemic, fewer cancer diagnoses were made and fewer treatments initiated, which contributed to increased mortality and adverse oncological outcomes (20–24).

Furthermore, differences in healthcare structures likely contributed to the observed findings. Data from a German comprehensive cancer center suggest that tertiary referral hospitals continued to treat a higher proportion of advanced and complex oncological cases during the pandemic. This can be explained, at least in part, by changes in referral patterns and the establishment of COVID-free treatment pathways, which enabled these centers to maintain surgical activity (25). Consequently, advanced-stage tumors became increasingly concentrated in such specialized institutions. This case-mix may also have contributed to the observed differences in postoperative survival between the study periods and should therefore be considered when interpreting the results. This interpretation is consistent with the findings of the present study, in which the majority of patients with higher UICC stages were treated in the referral center, supporting its role as a regional hub for complex gastrointestinal and endocrine oncological surgery. In addition to health system factors, patient-related aspects also played a significant role. During the COVID-19 pandemic, several studies reported a reduced utilization of healthcare services, partly driven by fear of SARS-CoV-2 infection as well as by efforts to minimize physical contact and healthcare exposure (26, 27).

The Kaplan–Meier analysis showed a trend toward shorter postoperative overall survival in patients operated on during the pandemic period, although the overall difference between study periods did not reach statistical significance (log-rank p = 0.066). Survival curves were largely comparable in the early postoperative phase, whereas a clear separation emerged over time, indicating shorter long-term survival in patients operated on during the pandemic period. This pattern suggests that differences between study periods became more apparent during longer follow-up, while immediate postoperative outcomes appeared comparable. However, the underlying mechanisms remain uncertain and cannot be attributed solely to pandemic-related changes in care. Survival probabilities in the pandemic cohort were numerically lower across all reported time points than in the pre-pandemic and post-pandemic cohorts.

Although the unadjusted Kaplan–Meier comparison showed only a statistical trend, multivariable Cox regression demonstrated an association between the pre-pandemic period and longer postoperative overall survival compared with the pandemic period after adjustment for age, sex, tumor stage, and resection site. These findings may reflect changes in oncological care pathways during the pandemic. However, differences in patient case-mix, referral patterns, unmeasured confounders, and causes of death may also have contributed to the observed survival differences. Several factors discussed in the literature may have contributed to the observed associations. In addition to delays in surgical treatment, multiple studies have described modifications in therapeutic strategies, including altered prioritization concepts, temporary adaptations of multimodal treatment pathways, and the increased use of non-surgical neoadjuvant therapies as bridging approaches in response to restricted healthcare resources (20). Furthermore, delays in cancer diagnosis and treatment initiation have repeatedly been associated with adverse oncological outcomes and disease progression, suggesting that interruptions in timely cancer care may have contributed to differences in oncological outcomes observed during the pandemic period (7–9, 28). Restrictions in outpatient care, delays in adjuvant therapies, and pandemic-related changes in clinical workflows could also have impaired continuity of oncological follow-up and subsequent treatment (20, 29–31).

This study has several limitations that must be considered when interpreting the findings. Given its retrospective design, the analysis is susceptible to information bias, as certain data elements may be incomplete, inaccurately recorded, or inconsistently documented across patients. Additionally, the analysis is based on data from three cooperating hospitals within a single regional network, which may limit the generalizability of the findings to other healthcare settings.

The inclusion of multiple gastrointestinal and endocrine tumor entities with distinct biological behavior, prognostic profiles, and treatment pathways represents an important limitation. Since the primary objective was to evaluate oncological surgical care at the healthcare system level, all eligible oncological resections were analyzed together. Therefore, survival analyses reflect the overall population of operated oncological patients rather than disease-specific survival patterns. Although resection site was included as an adjustment variable in the multivariable Cox regression model and exploratory site-specific analyses were performed, residual confounding related to tumor biology, stage distribution, and treatment pathways cannot be excluded. In addition, the concentration of complex oncological cases at the tertiary referral center may have influenced stage distribution, referral patterns, case-mix, and postoperative survival outcomes.

Furthermore, only patients undergoing oncological surgery were included. Consequently, the results cannot be generalized to all patients with cancer, as patients who did not undergo surgery due to advanced disease, frailty, altered treatment strategies, or other reasons were not captured. This may have introduced selection bias. Despite multivariable adjustment, residual confounding remains possible, as relevant factors influencing both treatment selection and survival were not consistently available across all participating hospitals. These include comorbidities, baseline performance status, neoadjuvant and adjuvant oncological therapies, treatment intensity, socioeconomic characteristics, SARS-CoV-2 infection status, and causes of death. Furthermore, relevant clinical variables such as UICC stage, resection status, and ASA classification were not available for all patients, which may have reduced the precision of risk adjustment. ASA classification was mainly available from the tertiary referral center and was therefore not included in the multivariable model due to incomplete availability across participating institutions. Information regarding diagnostic delays and treatment pathway intervals was also unavailable. In addition, different editions of the UICC staging system may have been applied during the study period according to contemporary clinical practice, representing a potential source of classification variability.

The shorter follow-up duration in the post-pandemic cohort reflects the predefined study period and represents administrative censoring rather than loss to follow-up. Therefore, long-term survival estimates for this cohort require cautious interpretation, particularly at later time points where the number of patients at risk decreases. Finally, the proportional hazards assumption of the Cox regression model was not formally assessed. Therefore, this methodological limitation should be considered when interpreting the regression estimates.

Despite these limitations, several methodological strengths should be acknowledged. The long observation period with consistently defined and equally timed comparison intervals allows the evaluation of temporal developments across different phases of the pandemic. The inclusion of three hospitals representing different levels of oncological care provides insight into surgical activity within a regional healthcare network. The large cohort size and inclusion of multiple gastrointestinal and endocrine resection sites contribute to a broad representation of oncological surgical practice. The use of Kaplan–Meier analyses and multivariable Cox regression models provides complementary information on postoperative overall survival. Nevertheless, these analyses should be interpreted within the limitations of the retrospective design and the available clinical variables. Furthermore, the endpoint of postoperative survival is objective and clearly defined, supporting the reliability of the outcome assessment.

This regional analysis demonstrated fluctuations in oncological surgical volume during individual COVID-19 pandemic waves, while the overall number of procedures was comparable across the three predefined study periods. Among operated patients, the unadjusted Kaplan–Meier analysis showed only a trend toward shorter postoperative overall survival during the pandemic period, whereas the multivariable Cox regression demonstrated a lower hazard of death in the pre-pandemic cohort compared with the pandemic cohort after adjustment for selected clinical variables. However, differences in hospital composition, tumor characteristics, missing data, and limited follow-up restrict causal attribution of these findings to the COVID-19 pandemic. These findings emphasize the importance of maintaining resilient and guideline-concordant oncological care pathways during future healthcare disruptions.

## Data Availability

The raw data supporting the conclusions of this article will be made available by the authors, without undue reservation.
