# Supplementary material for: Changes in oncological surgical volume and postoperative survival during the COVID-19 pandemic: a retrospective multicenter study in a regional German hospital network
Source: Front Oncol. 2026 Sep 7;16:1931801. doi: 10.3389/fonc.2026.1931801 (PMC13590334; doi:10.3389/fonc.2026.1931801)
Supplement: Supplementary file 1 [file DataSheet1.docx]

Supplementary Table S1: STROBE checklist for cohort studies

|  | | Item No | Recommendation | Page No |
| --- | --- | --- | --- | --- |
| **Title and abstract** | | 1 | (*a*) Indicate the study’s design with a commonly used term in the title or the abstract | 1 |
|  |  |  | (*b*) Provide in the abstract an informative and balanced summary of what was done and what was found | 1 |
| Introduction | | | | |
| Background/rationale | | 2 | Explain the scientific background and rationale for the investigation being reported | 3 |
| Objectives | | 3 | State specific objectives, including any prespecified hypotheses | 3 |
| Methods | | | | |
| Study design | | 4 | Present key elements of study design early in the paper | 4 |
| Setting | | 5 | Describe the setting, locations, and relevant dates, including periods of recruitment, exposure, follow-up, and data collection | 4 |
| Participants | | 6 | (*a*) Give the eligibility criteria, and the sources and methods of selection of participants. Describe methods of follow-up | 4 |
|  |  |  | (*b*) For matched studies, give matching criteria and number of exposed and unexposed | N/A |
| Variables | | 7 | Clearly define all outcomes, exposures, predictors, potential confounders, and effect modifiers. Give diagnostic criteria, if applicable | 5 |
| Data sources/ measurement | | 8* | For each variable of interest, give sources of data and details of methods of assessment (measurement). Describe comparability of assessment methods if there is more than one group | 4-5 |
| Bias | | 9 | Describe any efforts to address potential sources of bias | 5, 12-13 |
| Study size | | 10 | Explain how the study size was arrived at | 5 |
| Quantitative variables | | 11 | Explain how quantitative variables were handled in the analyses. If applicable, describe which groupings were chosen and why | 4-5 |
| Statistical methods | | 12 | (*a*) Describe all statistical methods, including those used to control for confounding | 5 |
|  |  |  | (*b*) Describe any methods used to examine subgroups and interactions | 5 |
|  |  |  | (*c*) Explain how missing data were addressed | 5 |
|  |  |  | (*d*) If applicable, explain how loss to follow-up was addressed | 5 |
|  |  |  | (*e*) Describe any sensitivity analyses | N/A |
| Results | | | |  |
| Participants | | 13* | (a) Report numbers of individuals at each stage of study—eg numbers potentially eligible, examined for eligibility, confirmed eligible, included in the study, completing follow-up, and analysed | 6-9 |
|  |  |  | (b) Give reasons for non-participation at each stage | 4 |
|  |  |  | (c) Consider use of a flow diagram | N/A |
| Descriptive data | | 14* | (a) Give characteristics of study participants (eg demographic, clinical, social) and information on exposures and potential confounders | 6-7 |
|  |  |  | (b) Indicate number of participants with missing data for each variable of interest | 7 |
|  |  |  | (c) Summarise follow-up time (eg, average and total amount) | 5 |
| Outcome data | | 15* | Report numbers of outcome events or summary measures over time | 8-10 |
| Main results | 16 | (*a*) Give unadjusted estimates and, if applicable, confounder-adjusted estimates and their precision (eg, 95% confidence interval). Make clear which confounders were adjusted for and why they were included | | 8,10 |
|  |  | (*b*) Report category boundaries when continuous variables were categorized | | 4-5 |
|  |  | (*c*) If relevant, consider translating estimates of relative risk into absolute risk for a meaningful time period | | N/A |
| Other analyses | 17 | Report other analyses done—eg analyses of subgroups and interactions, and sensitivity analyses | | Supplement S2-S12 |
| Discussion | | | | |
| Key results | 18 | Summarise key results with reference to study objectives | | 11,13 |
| Limitations | 19 | Discuss limitations of the study, taking into account sources of potential bias or imprecision. Discuss both direction and magnitude of any potential bias | | 12 |
| Interpretation | 20 | Give a cautious overall interpretation of results considering objectives, limitations, multiplicity of analyses, results from similar studies, and other relevant evidence | | 11-13 |
| Generalisability | 21 | Discuss the generalisability (external validity) of the study results | | 13 |
| Other information | | | | |
| Funding | 22 | Give the source of funding and the role of the funders for the present study and, if applicable, for the original study on which the present article is based | | 14 |

Supplementary Table S2: Characteristics of oncologic surgical procedures stratified by hospital

|  | **Hospital 1 (referral center)** | | | **Hospital 2** | | | **Hospital 3** | | |
| --- | --- | --- | --- | --- | --- | --- | --- | --- | --- |
| **Time period** | **pre-pandemic (n=672)** | **pandemic (n=726)** | **post-pandemic**  **(n=735)** | **pre-pandemic**  **(n=35)** | **pandemic (n=42)** | **post-pandemic**  **(n=31)** | **pre-pandemic**  **(n=245)** | **pandemic (n=172)** | **post-pandemic**  **(n=213)** |
| male | 393 (58.5) | 421 (58.0) | 389 (52.9) | 20 (57.1) | 22 (52.4) | 19 (61.3) | 131 (53.5) | 86 (50.0) | 131 (61.5) |
| female | 279 (41.5) | 305 (42.0) | 346 (47.1) | 15 (42.9) | 20 (47.6) | 12 (38.7) | 114 (46.5) | 86 (50.0) | 82 (38.5) |
| ***age*** |  |  |  |  |  |  |  |  |  |
| median (IQR) | 65 (54-75) | 64 (54-73) | 63 (53-73) | 68 (62-78) | 76 (68-79) | 72 (60-79) | 73 (63-81) | 72 (64-81) | 71 (61-80) |
| ***UICC stage (%)*** | *n=622* | *n=693* | *n=709* | *n=34* | *n=37* | *n=21* | *n=193* | *n=122* | *n=170* |
| 0 | 7 (1.1) | 11 (1.6) | 14 (2.0) | 0 (0.0) | 0 (0.0) | 0 (0.0) | 1 (0.5) | 2 (1.6) | 1 (1.4) |
| I | 173 (27.8) | 173 (25.0) | 204 (28.8) | 7 (20.6) | 15 (40.6) | 3 (14.3) | 53 (27.5) | 29 (23.8) | 45 (26.5) |
| II | 132 (21.2) | 153 (22.1) | 160 (22.5) | 7 (20.6) | 14 (37.8) | 5 (23.8) | 51 (26.4) | 39 (32.0) | 49 (28.8) |
| III | 144 (23.2) | 149 (21.5) | 138 (19.5) | 13 (38.2) | 6 (16.2) | 10 (47.6) | 58 (30.1) | 33 (27.0) | 53 (31.2) |
| IV | 166 (26.7) | 207 (29.8) | 193 (27.2) | 7 (20.6) | 2 (5.4) | 3 (14.3) | 30 (15.5) | 19 (15.6) | 22 (12.9) |
| ***resectability (%)*** | *n=600* | *n=661* | *n=667* | *n=29* | *n=36* | *n=27* | *n=221* | *n=157* | *n=184* |
| R0 | 501 (83.5) | 552 (83.5) | 540 (81.0) | 28 (96.6) | 34 (94.4) | 27 (100) | 187 (84.6) | 138 (87.9) | 164 (89.2) |
| R1 | 74 (12.3) | 80 (12.1) | 95 (14.2) | 1 (3.4) | 2 (5.6) | 0 (0.0) | 9 (4.1) | 9 (5.7) | 8 (4.3) |
| R2 | 14 (2.3) | 11 (1.7) | 16 (2.4) | 0 (0.0) | 0 (0.0) | 0 (0.0) | 6 (2.7) | 3 (1.9) | 0 (0.0) |
| RX | 11 (1.9) | 18 (2.7) | 16 (2.4) | 0 (0.0) | 0 (0.0) | 0 (0.0) | 19 (8.6) | 7 (4.5) | 12 (6.5) |
| ***ASA classification^1^ (%)*** | *n=669* | *n=725* | *n=735* |  |  |  |  |  |  |
| I | 32 (4.8) | 20 (2.8) | 24 (3.3) | n.a. | n.a. | n.a. | n.a. | n.a. | n.a. |
| II | 204 (30.5) | 203 (28.0) | 242 (32.9) | n.a. | n.a. | n.a. | n.a. | n.a. | n.a. |
| III | 404 (60.4) | 467 (64.4) | 444 (60.4) | n.a. | n.a. | n.a. | n.a. | n.a. | n.a. |
| IV | 28 (4.2) | 34 (4.7) | 24 (3.3) | n.a. | n.a. | n.a. | n.a. | n.a. | n.a. |
| V | 1 (0.1) | 1 (0.1) | 1 (0.1) | n.a. | n.a. | n.a. | n.a. | n.a. | n.a. |
| ***surgical intent*** |  |  |  |  |  |  |  |  |  |
| primary tumor resection | 619 (92.1) | 685 (94.4) | 692 (94.1) | 35 (100) | 42 (100) | 31 (100) | 235 (95.9) | 169 (98.3) | 210 (100) |
| metastasectomy | 53 (7.9) | 41 (5.6) | 43 (5.9) | 0 (0.0) | 0 (0.0) | 0 (0.0) | 10 (4.1) | 3 (1.7) | 3 (1.4) |
| ***surgical urgency*** |  |  |  |  |  |  |  |  |  |
| elective | 650 (96.7) | 709 (97.7) | 723 (98.4) | 35 (100) | 42 (100) | 31 (100) | 235 (100) | 172 (100) | 213 (100) |
| emergency | 22 (3.3) | 17 (2.3) | 12 (1.6) | 0 (0.0) | 0 (0.0) | 0 (0.0) | 10 (0.0) | 0 (0.0) | 0 (0.0) |
| ***resection site (%)*** |  |  |  |  |  |  |  |  |  |
| colon | 117 (17.4) | 83 (11.4) | 65 (8.8) | 19 (54.3) | 28 (66.6) | 19 (61.3) | 98 (40.0) | 81 (47.1) | 112 (52.6) |
| stomach | 35 (5.2) | 28 (3.9) | 31 (4.2) | 2 (5.7) | 2 (4.8) | 6 (19.5) | 23 (9.4) | 22 (12.8) | 11 (5.2) |
| oesophagus | 51 (7.6) | 71 (9.8) | 71 (9.7) | 1 (2.9) | 1 (2.4) | 0 (0.0) | 2 (0.8) | 1 (0.6) | 2 (0.9) |
| pancreas | 89 (13.3) | 94 (12.9) | 112 (15.2) | 0 (0.0) | 0 (0.0) | 0 (0.0) | 20 (8.2) | 7 (4.1) | 1 (0.5) |

| rectum | 72 (10.7) | 80 (11.0) | 30 (4.1) | 12 (34.2) | 8 (19.0) | 2 (6.4) | 59 (24.1) | 47 (27.3) | 73 (34.2) |
| --- | --- | --- | --- | --- | --- | --- | --- | --- | --- |
| thyroid | 204 (30.4) | 227 (31.3) | 300 (40.8) | 1 (2.9) | 2 (4.8) | 2 (6.4) | 8 (3.3) | 4 (2.3) | 0 (0.0) |
| other^2^ | 61 (9.0) | 102 (14.0) | 83 (11.3) | 0 (0.0) | 1 (2.4) | 2 (6.4) | 25 (10.1) | 7 (4.1) | 11 (5.2) |
| liver metastases | 43 (6.4) | 41 (5.7) | 43 (5.9) | 0 (0.0) | 0 (0.0) | 0 (0.0) | 10 (4.1) | 3 (1.7) | 3 (1.4) |

^1^ ASA classification was available only for Hospital 1, as this variable was not routinely documented in the electronic systems of Hospitals 2 and 3 during the study period. ^2^other= appendix, duodenum, gallbladder, ileum, liver, thymus, and soft tissue sarcoma. pre-pandemic period: 01.08.2017-29.02.2020. pandemic period: 01.03.2020-30.09.2022. post-pandemic period: 01.10.2022-30.04.2025. ASA=American Society of Anesthesiologists. IQR= interquartile range. UICC= Union for International Cancer Control.

Supplementary Tables S3–S13 and Figures S1-S11. Patient-based postoperative survival analyses stratified by hospital and resection site

**Postoperative survival analyses by hospital**

Supplementary Table S3: Kaplan–Meier survival probabilities at predefined postoperative time points across pre-pandemic, pandemic, and post-pandemic periods in patients from hospital 1 (referral center)

| **postoperative** **time point** | **pre-pandemic (n=623)** | **pandemic (n=646)** | **post-pandemic (n=652)** |
| --- | --- | --- | --- |
| 1 month | 97.3% | 97.0% | 96.7% |
| 6 months | 87.7% | 86.2% | 89.3% |
| 12 months | 78.4% | 75.0% | not estimated^1^ |
| 24 months | 67.6% | 63.8% | not estimated^1^ |
| 36 months | 60.0% | 56.7% | not estimated^1^ |

pre-pandemic (*01.08.2017-29.02.2020);* pandemic (*01.03.2020-30.09.2022)*; post-pandemic (*01.10.2022-30.04.2025*); Differences between groups were assessed by log-rank test (p=0.197). ^1^follow-up pending.

Supplementary Figure S1: Kaplan-Meier survival curves for patients in hospital 1 stratified by period. pre-pandemic *(01.08.2017-29.02.2020)* n= 623; pandemic *(01.03.2020-30.09.2022)* n= 646; post-pandemic *(01.10.2022-30.04.2025)* n= 652. Differences between groups were assessed by log-rank test (p=0.197). ^1^follow-up pending.

Supplementary Table S4: Kaplan–Meier survival probabilities at predefined postoperative time points across pre-pandemic, pandemic, and post-pandemic periods in patients from hospital 2

| **postoperative** **time point** | **pre-pandemic (n=35)** | **pandemic (n=41)** | **post-pandemic (n=30)** |
| --- | --- | --- | --- |
| 1 month | 94.0% | 92.5% | 96.6% |
| 6 months | 90.9% | 89.9% | 79.4% |
| 12 months | 90.9% | 86.9% | not estimated^1^ |
| 24 months | 90.9% | 80.7% | not estimated^1^ |
| 36 months | 85.2% | 76.5% | not estimated^1^ |

pre-pandemic (*01.08.2017-29.02.2020);* pandemic (*01.03.2020-30.09.2022)*; post-pandemic (*01.10.2022-30.04.2025*); Differences between groups were assessed by log-rank test (p=0.084). ^1^follow-up pending.

Supplementary Figure S2: Kaplan-Meier survival curves for patients in hospital 2 stratified by period. pre-pandemic *(01.08.2017-29.02.2020)* n= 35; pandemic *(01.03.2020-30.09.2022)* n= 41; post-pandemic *(01.10.2022-30.04.2025)* n= 30. Differences between groups were assessed by log-rank test (p=0.084). ^1^follow-up pending.

Supplementary Table S5: Kaplan–Meier survival probabilities at predefined postoperative time points across pre-pandemic, pandemic, and post-pandemic periods in patients from hospital 3

| **postoperative** **time point** | **pre-pandemic (n=221)** | **pandemic (n=153)** | **post-pandemic (n=202)** |
| --- | --- | --- | --- |
| 1 month | 97.4% | 98.2% | 100% |
| 6 months | 95.4% | 96.2% | 94.3% |
| 12 months | 92.4% | 92.8% | not estimated^1^ |
| 24 months | 89.0% | 91.1% | not estimated^1^ |
| 36 months | 85.6% | 82.7% | not estimated^1^ |

pre-pandemic (*01.08.2017-29.02.2020);* pandemic (*01.03.2020-30.09.2022)*; post-pandemic (*01.10.2022-30.04.2025*); Differences between groups were assessed by log-rank test (p=0.860). ^1^follow-up pending.

Supplementary Figure S3: Kaplan-Meier survival curves for patients in hospital 3 stratified by period. pre-pandemic *(01.08.2017-29.02.2020)* n= 221; pandemic *(01.03.2020-30.09.2022)* n= 153; post-pandemic *(01.10.2022-30.04.2025)* n= 202. Differences between groups were assessed by log-rank test (p=0.860). ^1^follow-up pending.

**Postoperative survival analyses by resection site**

Supplementary Table S6: Kaplan–Meier survival probabilities at predefined postoperative time points for patients with colon cancer across pre-pandemic, pandemic, and post-pandemic periods

| **postoperative** **time point** | **pre-pandemic (n=212)** | **pandemic (n=164)** | **post-pandemic (n=178)** |
| --- | --- | --- | --- |
| 1 month | 97.6% | 98.1% | 97.8% |
| 6 months | 90.3% | 91.4% | 89.5% |
| 12 months | 84.8% | 86.0% | not estimated^1^ |
| 24 months | 78.9% | 77.3% | not estimated^1^ |
| 36 months | 75.3% | 70.8% | not estimated^1^ |

pre-pandemic (*01.08.2017-29.02.2020);* pandemic (*01.03.2020-30.09.2022)*; post-pandemic (*01.10.2022-30.04.2025*); Differences between groups were assessed by log-rank test (p=0.523). ^1^follow-up pending.

Supplementary Figure S4: Kaplan-Meier survival curves for patients with colon cancer stratified by period. pre-pandemic *(01.08.2017-29.02.2020)* n= 212; pandemic *(01.03.2020-30.09.2022)* n= 164; post-pandemic *(01.10.2022-30.04.2025)* n= 178. Differences between groups were assessed by log-rank test (p=0.523). ^1^follow-up pending.

Supplementary Table S7: Kaplan–Meier survival probabilities at predefined postoperative time points for patients with stomach cancer across pre-pandemic, pandemic, and post-pandemic periods

| **postoperative** **time point** | **pre-pandemic (n=59)** | **pandemic (n=47)** | **post-pandemic (n=46)** |
| --- | --- | --- | --- |
| 1 month | 98.0% | 94.5% | 100% |
| 6 months | 90.6% | 85.2% | 82.0% |
| 12 months | 75.8% | 67.4% | not estimated^1^ |
| 24 months | 72.2% | 50.5% | not estimated^1^ |
| 36 months | 72.2% | 36.1% | not estimated^1^ |

pre-pandemic (*01.08.2017-29.02.2020);* pandemic (*01.03.2020-30.09.2022)*; post-pandemic (*01.10.2022-30.04.2025*); Differences between groups were assessed by log-rank test (p=0.074). ^1^follow-up pending.

Supplementary Figure S5: Kaplan-Meier survival curves for patients with stomach cancer stratified by period. pre-pandemic *(01.08.2017-29.02.2020)* n= 59; pandemic *(01.03.2020-30.09.2022)* n= 47; post-pandemic *(01.10.2022-30.04.2025)* n= 46. Differences between groups were assessed by log-rank test (p=0.074). ^1^follow-up pending.

Supplementary Table S8: Kaplan–Meier survival probabilities at predefined postoperative time points for patients with oesophageal cancer across pre-pandemic, pandemic, and post-pandemic periods

| **postoperative** **time point** | **pre-pandemic (n=50)** | **pandemic (n=69)** | **post-pandemic (n=69)** |
| --- | --- | --- | --- |
| 1 month | 89.8% | 95.7% | 97.0% |
| 6 months | 72.3% | 79.0% | 82.0% |
| 12 months | 58.4% | 63.1% | not estimated^1^ |
| 24 months | 45.9% | 49.7% | not estimated^1^ |
| 36 months | 32.8% | 38.7% | not estimated^1^ |

pre-pandemic (*01.08.2017-29.02.2020);* pandemic (*01.03.2020-30.09.2022)*; post-pandemic (*01.10.2022-30.04.2025*); Differences between groups were assessed by log-rank test (p=0.398). ^1^follow-up pending.

Supplementary Figure S6: Kaplan-Meier survival curves for patients with oesophageal cancer stratified by period. pre-pandemic *(01.08.2017-29.02.2020)* n= 50; pandemic *(01.03.2020-30.09.2022)* n= 69; post-pandemic *(01.10.2022-30.04.2025)* n= 69. Differences between groups were assessed by log-rank test (p=0.398). ^1^follow-up pending.

Supplementary Table S9: Kaplan–Meier survival probabilities at predefined postoperative time points for patients with pancreatic cancer across pre-pandemic, pandemic, and post-pandemic periods

| **postoperative** **time point** | **pre-pandemic (n=104)** | **pandemic (n=100)** | **post-pandemic (n=105)** |
| --- | --- | --- | --- |
| 1 month | 97.0% | 94.9% | 94.9% |
| 6 months | 86.4% | 82.0% | 85.9% |
| 12 months | 70.3% | 62.7% | not estimated^1^ |
| 24 months | 50.2% | 44.1% | not estimated^1^ |
| 36 months | 37.7% | 36.7% | not estimated^1^ |

pre-pandemic (*01.08.2017-29.02.2020);* pandemic (*01.03.2020-30.09.2022)*; post-pandemic (*01.10.2022-30.04.2025*); Differences between groups were assessed by log-rank test (p=0.614). ^1^follow-up pending.

Supplementary Figure S7: Kaplan-Meier survival curves for patients with pancreatic cancer stratified by period. pre-pandemic *(01.08.2017-29.02.2020)* n= 104; pandemic *(01.03.2020-30.09.2022)* n= 100; post-pandemic *(01.10.2022-30.04.2025)* n= 105. Differences between groups were assessed by log-rank test (p=0.614). ^1^follow-up pending.

Supplementary Table S10: Kaplan–Meier survival probabilities at predefined postoperative time points for patients with rectal cancer across pre-pandemic, pandemic, and post-pandemic periods

| **postoperative** **time point** | **pre-pandemic (n=120)** | **pandemic (n=112)** | **post-pandemic (n=92)** |
| --- | --- | --- | --- |
| 1 month | 97.4% | 97.3% | 100% |
| 6 months | 94.8% | 86.8% | 96.2% |
| 12 months | 89.1% | 80.4% | not estimated^1^ |
| 24 months | 83.0% | 70.7% | not estimated^1^ |
| 36 months | 74.8% | 64.7% | not estimated^1^ |

pre-pandemic (*01.08.2017-29.02.2020);* pandemic (*01.03.2020-30.09.2022)*; post-pandemic (*01.10.2022-30.04.2025*); Differences between groups were assessed by log-rank test (p=0.054). ^1^follow-up pending.

Supplementary Figure S8: Kaplan-Meier survival curves for patients with rectal cancer stratified by period. pre-pandemic *(01.08.2017-29.02.2020)* n= 120; pandemic *(01.03.2020-30.09.2022)* n= 112; post-pandemic *(01.10.2022-30.04.2025)* n= 92. Differences between groups were assessed by log-rank test (p=0.054). ^1^follow-up pending.

Supplementary Table S11: Kaplan–Meier survival probabilities at predefined postoperative time points for patients with thyroid cancer across pre-pandemic, pandemic, and post-pandemic periods

| **postoperative** **time point** | **pre-pandemic (n=192)** | **pandemic (n=215)** | **post-pandemic (n=269)** |
| --- | --- | --- | --- |
| 1 month | 99.3% | 98.2% | 98.6% |
| 6 months | 96.5% | 92.2% | 96.5% |
| 12 months | 95.5% | 88.6% | not estimated^1^ |
| 24 months | 89.4% | 84.8% | not estimated^1^ |
| 36 months | 86.2% | 82.4% | not estimated^1^ |

pre-pandemic (*01.08.2017-29.02.2020);* pandemic (*01.03.2020-30.09.2022)*; post-pandemic (*01.10.2022-30.04.2025*); Differences between groups were assessed by log-rank test (p=0.270). ^1^follow-up pending.

Supplementary Figure S9: Kaplan-Meier survival curves for patients with thyroid cancer stratified by period. pre-pandemic *(01.08.2017-29.02.2020)* n= 192; pandemic *(01.03.2020-30.09.2022)* n= 215; post-pandemic *(01.10.2022-30.04.2025)* n= 269. Differences between groups were assessed by log-rank test (p=0.270). ^1^follow-up pending.

Supplementary Table S12: Kaplan–Meier survival probabilities at predefined postoperative time points for patients with other cancer sites across pre-pandemic, pandemic, and post-pandemic periods

| **postoperative** **time point** | **pre-pandemic (n=88)** | **pandemic (n=85)** | **post-pandemic (n=80)** |
| --- | --- | --- | --- |
| 1 month | 96.3% | 98.8% | 92.8% |
| 6 months | 86.4% | 94.8% | 83.9% |
| 12 months | 76.1% | 83.5% | not estimated^1^ |
| 24 months | 65.3% | 71.4% | not estimated^1^ |
| 36 months | 56.8% | 58.9% | not estimated^1^ |

pre-pandemic (*01.08.2017-29.02.2020);* pandemic (*01.03.2020-30.09.2022)*; post-pandemic (*01.10.2022-30.04.2025*); Differences between groups were assessed by log-rank test (p=0.817). ^1^follow-up pending.

Supplementary Figure S10: Kaplan-Meier survival curves for patients with other cancer sites stratified by period. pre-pandemic *(01.08.2017-29.02.2020)* n= 88; pandemic *(01.03.2020-30.09.2022)* n= 85; post-pandemic *(01.10.2022-30.04.2025)* n= 80. Differences between groups were assessed by log-rank test (p=0.817). ^1^follow-up pending.

Supplementary Table S13: Kaplan–Meier survival probabilities at predefined postoperative time points for patients with liver metastases across pre-pandemic, pandemic, and post-pandemic periods

| **postoperative** **time point** | **pre-pandemic (n=54)** | **pandemic (n=48)** | **post-pandemic (n=45)** |
| --- | --- | --- | --- |
| 1 month | 97.9% | 93.6% | 95.0% |
| 6 months | 83.1% | 77.6% | 82.5% |
| 12 months | 77.9% | 66.0% | not estimated^1^ |
| 24 months | 63.9% | 61.3% | not estimated^1^ |
| 36 months | 61.0% | 51.4% | not estimated^1^ |

pre-pandemic (*01.08.2017-29.02.2020);* pandemic (*01.03.2020-30.09.2022)*; post-pandemic (*01.10.2022-30.04.2025*); Differences between groups were assessed by log-rank test (p=0.699). ^1^follow-up pending.

Supplementary Figure S11: Kaplan-Meier survival curves for patients with liver metastases stratified by period. pre-pandemic *(01.08.2017-29.02.2020)* n= 54; pandemic *(01.03.2020-30.09.2022)* n= 48; post-pandemic *(01.10.2022-30.04.2025)* n= 45. Differences between groups were assessed by log-rank test (p=0.699). ^1^follow-up pending.
